# Seasonal variations of microplastic in sediment, *Chironomus* sp. larvae, and chironomid tubes in two wastewater sites in Sohag Governorate, Egypt

**DOI:** 10.1007/s11356-023-30855-4

**Published:** 2023-11-27

**Authors:** Azza M. Khedre, Somaia A. Ramadan, Ali Ashry, Mohamed Alaraby

**Affiliations:** 1 Group of Entomology and Environmental Toxicology, Department of Zoology, Faculty of Science, Sohag University, Sohag, 82524 Egypt; 2 Group of Mutagenesis, Department of Genetics and Microbiology, Faculty of Biosciences, Universitat Autònoma de Barcelona, Cerdanyola del Vallès, Barcelona, Spain

**Keywords:** Microplastic, Wastewater, Sediment, *Chironomus* sp., Chironomid tube

## Abstract

Microplastic (MP) contamination is an acknowledged global problem that poses a severe risk to aquatic ecosystem biota. Nevertheless, little is known about their prevalence in animal construction. The main objective of our study was to reduce the gap information of seasonal abundance, distribution, composition, and risk assessment of MP contamination. The concentrations of MPs in sediment, *Chironomus* sp. larvae, and their tubes were found to be higher in site 2 (S2) than in site 1 (S1) during the four seasons of the year. However, MP concentrations ranged from 312 ± 64.7 to 470 ± 70 items/kg dry weight, 0.79 ± 0.16 to 1.1 ± 0.3 particles/individual, and 0.5 ± 0.04 to 0.9 ± 0.04 particles/tube in sediment, *Chironomus*, and chironomid tubes, respectively. Blue and red polyester fibers are the most dominant MPs which are distributed in sediment, *Chironomus*, and chironomid tubes. The length of the dominant fiber accumulates in *Chironomus*, and their tubes are highly varied compared to that of the substrate. Additionally, we found that the mean number of MPs/individual larvae in the fourth instar was significantly higher than that in the second instar. Risk indicators for the environment, polymer risk assessment, and pollution load were estimated, where they were higher in S2 than in S1 correlated to MPs abundance and polymer type. The seasonal fluctuation in MP concentration, characterization, and risk in the two sites could depend on the amount of sewage effluent discharged into the wastewater treatment plants (WWTPs), which was reflected by *Chironomus* sp. larvae. Therefore, further research should be done to adopt the applicability of *Chironomus* as MP bioindicators in various freshwater environments throughout the world.

## Introduction

MP contamination has been recorded all over the world, from the poles to the equator, and from the ocean’s surface to the deepest abyss (Rochman et al. 2015; Blettler et al. 2018; Eerkes-Medrano and Thompson 2018; Peng et al. 2018a, b; Li et al. 2018; Mendoza and Balcer 2019; Tursi et al. 2022), and in the different media (Dobaradaran et al. 2018; Akhbarizadeh et al. 2020a, 2020b, 2021a, 2021b; Takdastan et al. 2021; De-la-Torre et al. 2022a, 2022b, 2023a, 2023b; Hajiouni et al. 2022; Kashfi et al. 2022, 2023; Mohammadi et al. 2022a, 2022b, 2023; Pizarro-Ortega et al. 2022; Pourfadakari et al. 2022; Cabrejos-Cardeña et al. 2023; Niari et al. 2023). MPs are particles of plastic smaller than 5 mm (Qu et al. 2023). They are transferred from terrestrial ecosystems via biotic and abiotic mechanisms, or they reach the aquatic environment directly through substances that contain MPs (Ory et al. 2017; Li et al. 2018). After that, MPs are further classified as primary MPs (plastic beads used in air blasting and cosmetic items) or secondary MPs (plastic fragments made from larger plastic particles) (Sharma et al. 2023). According to Burns et al. (2018) and Khedre et al. (2023a, b), MPs may be categorized into several form categories with names such as fragments, fibers, films, foam, and beads. The physical features of MPs, including density, shape, and size, may affect how they move across various environments and how they disperse (Hartmann et al. 2019). Stormwater runoff, industrial waste, and household rubbish are just a few of the pathways via which MPs may enter an aquatic ecosystem (Horton and Dixon 2018; Nel and Froneman 2018). Based on Li et al. (2018), the mean abundance of MPs in freshwater systems varied from almost none to several million pieces per cubic meter. Currently, there is exponential growth of MP research around the world since the first detection made around the coast of New Zealand in 1977 by Gregory (1977). However, there are very few studies on the existence of MPs in Africa.

Most of the research has classified each sampling site based on the principal human activities that qualitatively analyze the source of MPs (Lin et al. 2022). For instance, if research believes that a sample location having a high level of industrialization is typical of industrial districts, additional analysis will thus link industrial activities to the high MP content in the area surrounding the sampling site. However, this attribution is highly dubious since it ignores the temporal and hydrological aspects involved in MP transport via freshwater ecosystems (Klein et al. 2015). Residences may be found in highly industrialized areas. Furthermore, wastewater treatment plants (WWTPs) are well recognized as point sources of MPs because large volumes of MP-containing effluents are continually discharged (Grbić et al. 2020), even though the majority of the MPs are removed from the influents (Talvitie et al. 2017). Thus, WWTPs are frequently associated with significant levels of MP contamination. Recent research, for example, discovered greater MP concentrations in WWTP effluents compared to those in a reference location (Magnusson and Norén 2014).

Since MPs are readily observed in the internal tissues of animals (Prata et al. 2022), here, we use the term “internal MPs” to distinguish those MPs in either the digestive systems or internal organs of organisms from those distributed in the environment (environmental MPs). In general, there are much fewer studies on internal MPs (Lin et al. 2022) since the necessary processing steps become very complicated when comparing MPs sampled from water or sediment. However, we believe that there are both costs and benefits to investigating internal MPs.

Internal MPs also provide a longitudinal picture of the contamination of the environment with MPs. Throughout their life cycle, midge larvae are likely to accumulate MPs (Ziajahromi et al. 2018). When continuous sampling is not feasible, investigations of internal MPs offer a long-term picture of local MPs’ pollution. Because midge larvae have an intrinsic feeding strategy (i.e., they ingest MPs unintentionally with food), prior research has indicated that the quantity of MPs in sediments is related to the abundance of MPs in midge larvae (Nel et al. 2018). As an alternative, several studies have collected MPs in the environment utilizing a one-time sample technique, which may provide inaccurate information on the abundance of MPs because it only provides a snapshot of their contamination at that moment (Naji et al. 2017; Wagner and Lambert 2018). For instance, collecting MPs from the ocean’s surface at a particular moment in time was insufficient to assess the level of MP pollution since there was no MP buildup (Cheung et al. 2019). Studies on the temporal and geographical MPs distributions in the environment are accessible (Xia et al. 2021; Fan et al. 2022), but few studies specifically address MPs detected in living organisms.

In addition to ingesting, animals may integrate MPs into the structures they build. For instance, the marine polychaete *Gunnarea gaimardi* (de Quatrefages 1848) fixes MP particles in a biological structure by incorporating them into its habitat (Nel and Froneman 2018). Additionally, MPs may be included in the larval cases (biological structures) produced by a variety of epibenthic insects, including freshwater caddis fly (Trichoptera) species (Ehlers et al. 2019). As a result, larval cases made by aquatic insects may be used as bioindicators for MP evaluations of freshwater systems. Chironomid tubes are comparable biological formations found in watery settings. Therefore, to determine whether freshwater MPs might be absorbed into chironomid tubes, we examined whether MPs were present in those tubes and what qualities (shape, polymer type, color, and size) they possessed. So, this study provides a quantitative comparison of the spatiotemporal parameters affecting the variation in MPs dominance in WWTPs environment. Moreover, it quantifies the combined effects of seasonality and anthropogenic activities on internal MP concentrations and estimates the contributions of factors affecting internal MP pollution in two wastewater sites in Egypt, as well as studying the importance of chironomid tubes as indicators for MP freshwater pollution.

In Egypt, no data is available on the seasonal occurrence, characterization, and risk of MP pollution in sediment, water, or freshwater insects. Additionally, this highlights the use of chironomid tubes to reflect MP buildup in the aquatic environment. Two wastewater sites in the Sohag Governorate were chosen to perform this investigation. Every day, a considerable volume of wastewater is dumped there. Furthermore, wastewater sediment can be used as soil fertilizer, leading MPs to be transported to agricultural land surfaces, which is of major concern. As a result, the current study aims to (i) quantify the concentration of MPs in the current wastewater basin’s sediment, *Chironomus* sp. larvae, and their tubes throughout the four seasons of the year to determine possible MPs risks in the two wastewater sites; (ii) answer the following questions: (a) whether the MPs loads in *Chironomus* sp. larvae and their tubes differ according to the different aquatic system differences in plastic pollution level; (b) whether the MPs loads in *Chironomus* sp. larvae differ according to the developmental stage and size; and (c) finally, whether *Chironomus* sp. larvae and their tubes can be used as qualitative and quantitative bioindicators for MPs in aquatic ecosystems; (iii) to identify shapes, colors, size, and polymeric characteristics of MPs extracted from water, sediment, and aquatic insects; and (IV) to assess the potential risks of MPs through multiple indices. In addition, offering the required knowledge of MP contamination in Sohag governorate to the policymaker and stockholders will encourage them to take the necessary actions for improving plastic waste management.

## Materials and methods

### Study area

**Fig. 1:**
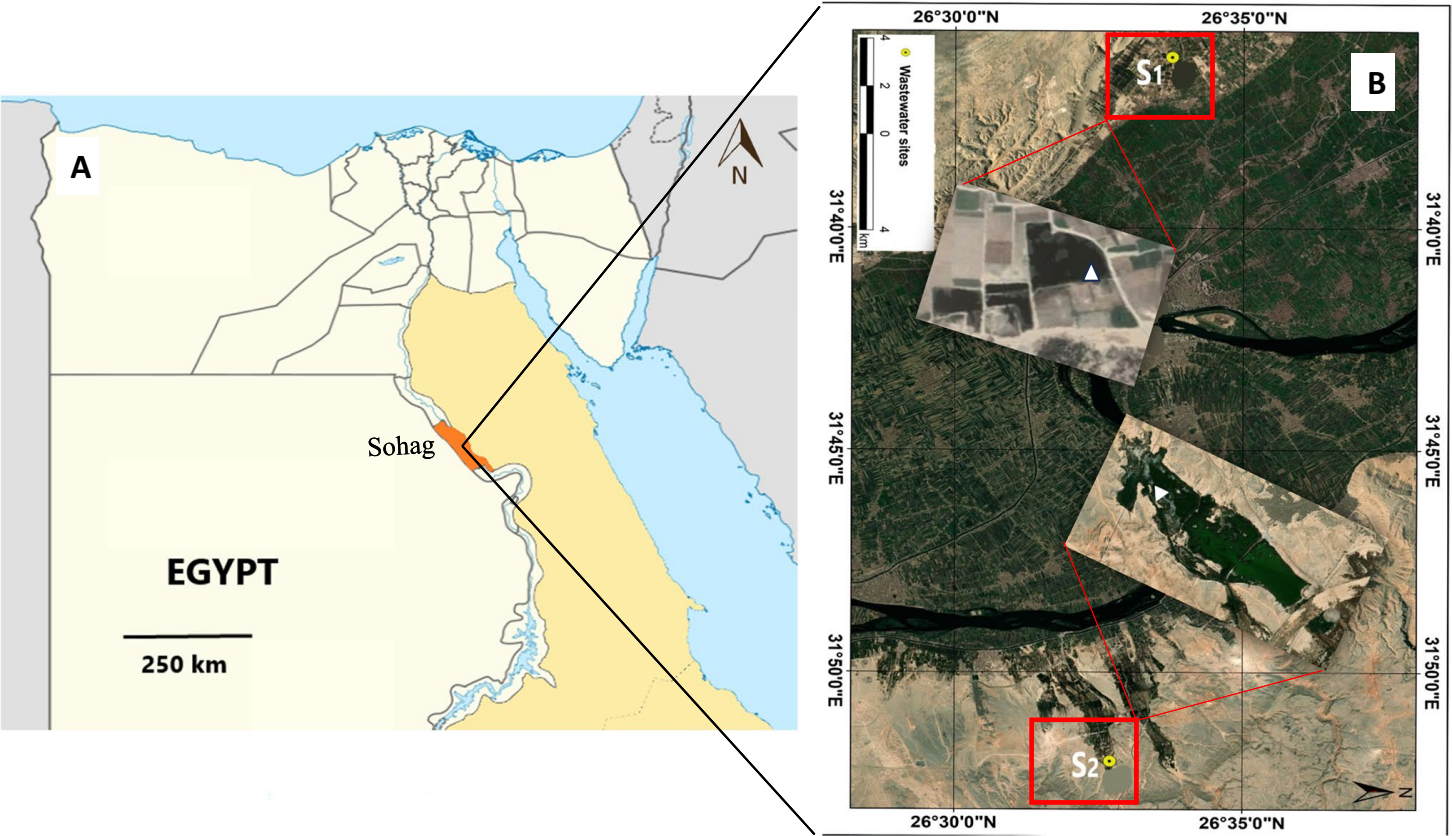
Egypt map showing Sohag Governorate (**A**). Google Earth photo showing the collecting sites (**B**)

The sampling area is in Sohag City, which is in Upper Egypt, in the midst of the Nile Valley (approximately 125 km long). Sohag stretches from the southernmost point of Assiut Governorate, located at latitude 26° 57′ N, to the northernmost point of Qena Governorate, located at latitude 26° 07′ N. Between longitudes 31° 20′ and 32° 14′ E, it is enclosed (Fig. 1A). The research location is in a dry region of North Africa, which is known for its scorching summers, moderate winters, and scant rainfall. Except for the regions where there are communities, the whole valley is mostly utilized for agricultural purposes. Newly cultivated margins delineate the valley’s eastern and western flanks. Land reclamation initiatives, new urban communities, industrial zones, and wastewater disposal sites in desert zones are some of the kinds of development in the area. There are sizable plants for the textile, soft drink, and sugar sectors nearby. The industries of macaroni, sweets, onion drying, and oil dehydration are all represented by other small private firms in the study region. In Sohag Governorate, two sites have been designated for wastewater disposal. One is in the western plateau and named the West wastewater treatment plant (S1) and the other is in the east plateau named the East wastewater treatment plant (S2), which were selected for sampling (Fig. 1B) in the winter (January), summer (July), spring (April), and autumn (October) of 2022.

### Description of the collection sites

Approximately 10 km west of Sohag city, Site 1 (S1) is situated at 26° 33′ 54″ North and 31° 36′ 54″ East. It is situated in the desert region between the agricultural floodplain to the east and the Eocene limestone plateau to the west. The basin is approximately 3.786 km^2^ in size and has a rectangular shape. It has an average depth of 1 m and is a shallow ecology. The basin is impacted by several sources of pollution, including the ongoing discharge of wastewater from local WWTPs, human activities brought on by the expansion of adjacent agriculture, the care of animals, and sewage flow from nearby settlements. Large amounts of algae are present throughout the basin, and the water movement is so sluggish that it may be termed stagnant water. At the boundaries, various plant patches could be seen. The effluent is turbid and filthy gray in color and has a fecal or rotting stench throughout the four seasons. The gravel at the bottom of the basin is often dark in color.

Site 2 (S2) is a location in the lowland desert south of the EL cola wastewater project, which is situated at a latitude of 26° 33′ 04′′ N and longitude of 31° 50′ 55′′ E, approximately 14.6 km east of Sohag city. On a surface of approximately 4.4 km^2^, this location has a number of irregular basins. In the whole area, a basin of approximately 1.27 km^2^ was selected for sampling. Low levels of vegetation are reflected by a few isolated plant patches around the basin’s edges. The level of the water, which is approximately 1.5 m, appears to remain still. Wastewater from neighboring WWTPs has been continuously discharged into the basin. This location appears to have few human activities, most of which are brought on by the existence of a few wooden trees. Additionally, the basin has been exposed to industrial waste because an industrial district (El-Kawser) is close to the current location.

### Sample collection

#### Sediment sampling

Through the four seasons of 2022, samples of sediment were taken from the two wastewater sampling sites (S1 and S2). Fifteen sediment samples, each weighing approximately 2 kg, were taken from the top sediment (0–5 cm depth) of each site at five randomly chosen points (three samples were taken from each point) using a stainless-steel spoon. The samples were subsequently held in glass containers in the lab at a temperature of − 4 °C to protect them from external particle contamination (sample collection was followed by a quick closure of the glass containers to prevent contamination from the air).

#### Chironomus sp. larvae and their tubes sampling

Chironomid larvae are the dominant genus present in the top layer of sediment in degraded habitats due to their tendency for hypoxic aquatic environments (Bere et al. 2016; Nhiwatiwa et al. 2017; Berezina et al. 2022). They deposit feeders and feed on detritus, and their associated bacteria and fungi settle in the sediment (Bertin et al. 2014). They commonly build tubes for protection (Scherer et al. 2017).

*Chironomus* sp. larvae were collected from the two investigated sites using a pond net (200-µm mesh size; 0.30-m aperture) at identical sediment collection points by immersing the hand net against the current while sweeping and kicking in the sediment. *Chironomus* sp. larvae were detected on an identification tray, and the samples were promptly preserved in 70% alcohol in 100-mL screw-capped glass vials to prevent the ejection of gut contents, which might influence the MP estimate, as described by Nel et al. (2018). The chironomid tubes were taken from sediment samples and stored in a deep freezer to extract MPs.

### Laboratory analysis

#### Extraction of MPs from wastewater sediment

Bagheri et al. (2020) approach was modified somewhat to extract the MPs from sediment. The sediment samples were placed in spick-and-span glass jars, and they were then dried for 48 h in an oven set to 60 °C. In an uncontaminated 1-L beaker, 100 g of each dry sediment sample was placed. MPs were isolated from denser natural particles using a density separation approach. Before adding a freshly hypersaline solution of NaCl/NaI (0.5 g/cm^3^) to the sediment samples, 30 mL of 30% H_2_O_2_ was added to each beaker to assist breakdown of any organic matter that may have been present (Zhou et al. 2018) (all the materials were purchased from Sigma-Aldrich (Ontario, Canada)). Then, the beakers were shaken at 200 rpm for 2 days on an open-air, dual-action shaker table (OS-2000, JEIOTECH, Korea) to separate any MPs. The floating supernatants were moved to a second beaker and allowed to settle for 24 h. After being filtered using 0.45-µm filter paper to collect all MP particles, they were further preserved for microscopic examination. To ensure that all of the MPs had been extracted from the sediment sample, all of the aforementioned procedures were repeated several times.

#### Preparation of Chironomus sp. larvae and their tubes

*Chironomus* sp. was isolated and identified using keys by De Moor et al. (2003). Five *Chironomus* subsamples were obtained from each sample site to determine the presence of MPs. Each subsample consisted of ten fourth instar similar-sized *Chironomus* sp. individuals.

To prepare chironomid tubes for MP extraction, metal forceps were used to remove the larvae from their tubes. To proceed, the tubes were placed in glass Petri dishes and then separated into 5 samples (each sample including 10 tubes). Each sample’s wet weight was calculated. To avoid cross-contamination of the chironomid tubes with MPs, we meticulously washed our forceps between samples. Finally, we promptly covered all the Petri dishes containing chironomid tubes with aluminum foil to prevent airborne MP contamination.

#### MPs extraction from Chironomus sp. larvae and their tubes

Clean glass test tubes were used to hold each subsample of *Chironomus* sp. larvae. Each test tube contained 20 ml of H_2_O_2_ (35% V/V) and was shaken at 200 rpm for 12 h to allow for reaction (Windsor et al. 2019). The *Chironomus* sp. remnants were then vacuum filtered using 0.45-µm filter paper before being put in a clean petri dish for further investigation. Each filter was examined under a stereomicroscope to visually identify and count MP particles. Chironomid tubes were also processed according to the steps described above.

#### MPs ingestion throughout the development of Chironomus sp. larvae

To test how the developmental stage affects MP uptake, two different instars of *Chironomus* sp. larvae were selected (second (L2) and fourth (L4)) to analyze the correlation between body size (related to the morphological characteristics) in each instar and their mean MPs content. One hundred randomly selected larvae from each instar were obtained from the field collection in the summer season at S2. They were divided into ten replicates, each containing ten individuals. The wet weight of each instar individual was determined. Additionally, measurements of the body length, head capsule length, and width were detected. The ten replicates of each instar were analyzed as described before, and the corresponding number, size, and shape of ingested MP particles were determined.

#### MPs identification and characterization

Using a dissecting microscope with a digital camera (Carl Zeiss Suzhou Co.), all MPs were counted visually. MP particle shapes and colors were also identified and photographed. All MP measurements (diameter and length) were measured using the ImageJ program (version 1.53f, available at https://imagej.net/ij/). ATR-FTIR spectroscopy (Alpha Bruker Platinum, 1–211-6353) was performed on a zinc slender crystal with an incidence angle of 45 ± 15 and a scan period of 560 s (24 s) with a resolution of 4 cm^−1^ (range, 4000–400 cm^−1^) to identify the chemical composition of MPs. The experiment used MP particles of various colors and shapes. The data were modified using the OPUS program (Bruker Optics GmbH). The polymer type was established by comparing the obtained spectra to known reference spectra (Primpke et al. 2018).

#### Experiment quality assurance

Samples were always kept sealed in a vial or Petri dish to prevent contamination, except when suspicious plastics were selected. The experimenters used no plastic items and were outfitted in cotton lab coats and gloves. Before usage, all containers were washed with Milli-Q water. Prior to use, all solutions utilized in the study were passed through three filters. To ensure that there was no contamination from the lab environment, three procedural blanks were performed. Throughout the investigation, no MP particles were found in the blanks. Three Petri dishes were set up near the workstation for a day to collect airborne particles to calculate the amount of contamination that was airborne. The results of this investigation were not considerably impacted by procedural contamination because we only collected one cotton fiber sample.

### MPs risk assessment

#### Pollution load index (PLI)

The following equations (Wang et al. 2020) were used to generate the pollution load index (PLI), which was used to quantify the risk of MPs contamination (Kasamesiri et al. 2023).

$$CFj= {~}^{Ci}\!\left/ \!{~}{Co}\right.$$

$$PLIj=\sqrt{CFj}$$

where $Ci$ is the MP concentration at sample site j and $Co$ is the background MP concentration. The reference values for MPs were adopted according to worldwide records for sediments (1.79 items/kg DW) (Guo et al. 2021). $PLIj$ was divided into four degrees of pollution by Guo et al. (2021).

#### Polymer risk assessment index

Li et al. (2020) calculated the polymer risk index (H) as follows:

$$H=\sum_{n=1}^{n}PnSn$$

where $Pn$ is the proportion of each polymer type at each sample site and $Sn$ is the polymer hazard score calculated by Lithner et al. (2011), with PP = 4, PES = 4, and PE = 11. Lithner et al. (2011) and Guo et al. (2021) divided H into four levels: level I, < 10; level II, 10–100; level III, 100–1000; and level IV, > 1000.

#### Potential ecological risk index (RI)

$RI$ Has been used to assess the ecological and toxicological consequences of MPs (Peng et al. 2018a, b; Ranjani et al. 2021).

$$Tj={~}^{H}\!\left/ \!{~0}_{Ci}\right.$$

$$RI=TjxCFj$$

$Tj$ denotes the toxicity coefficient of MPs. Guo et al. (2021) identified five contamination thresholds for $RI$ which are as follows: level I is less than 150, level II is 150–300, level III is 300–600, level IV is 600–1200, and level V is more than 1200.

### Statistical analysis

The main characteristics of MP concentration in sediment, *Chironomus* sp. larvae, and their tubes collected from various seasons and sites were described using descriptive statistics (mean and standard deviation SD), which were then submitted to one-way ANOVA (analysis of variance). Differences between means were deemed significant when *P* < 0.05. Using a *χ*2 test, it was possible to compare the relative proportion of MP lengths in sediment and *Chironomus* sp. larvae. The connection between the independent factors and the dependent variables was examined using univariate regression and Pearson rank correlation analysis. Using IBM SPSS (ver. 22, IBM Corp., Armonk, NY, USA), data analysis was carried out.

## Results

### Seasonal abundance and characterization of MPs in sediment

**Fig. 2:**
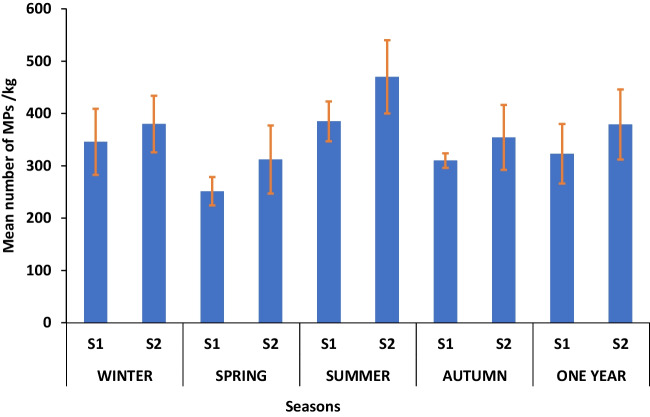
Mean seasonal abundance of MPs in the sediment of the two sites of wastewater

MPs were detected in all sediment samples with a 100% detection rate. Figure 2 shows the seasonal distribution of MPs in the sediment of the two wastewater sites, where the abundance of MPs in S1 and S2 during the winter season was 345.8 ± 63 and 380 ± 54 items/kg, respectively, with a mean value of 363 ± 24 items/kg. The spring season MP abundances in S1 and S2 were 251.4 ± 27.2 and 312 ± 64.7 items/kg, respectively, with a mean value of 281 ± 43 items/kg. The summer season MP abundance in S1 and S2 was 385.2 ± 38.2 and 470 ± 70 items/kg, respectively, with a mean value of 427 ± 60 items/kg. During the autumn season, the abundance of MPs in S1 and S2 was 310 ± 84 and 354.2 ± 62 items/kg, respectively, with a mean value of 332 ± 31 items/kg. Statistically, the abundance values varied significantly from season to season at the two sampling sites (*P* < 0.05), and the abundance of MPs was significantly higher in summer than in the other seasons (*P* < 0.05). Additionally, the MP abundance was significantly higher in S2 than in S1 in all seasons of the year (*P* < 0.05).

**Fig. 3:**
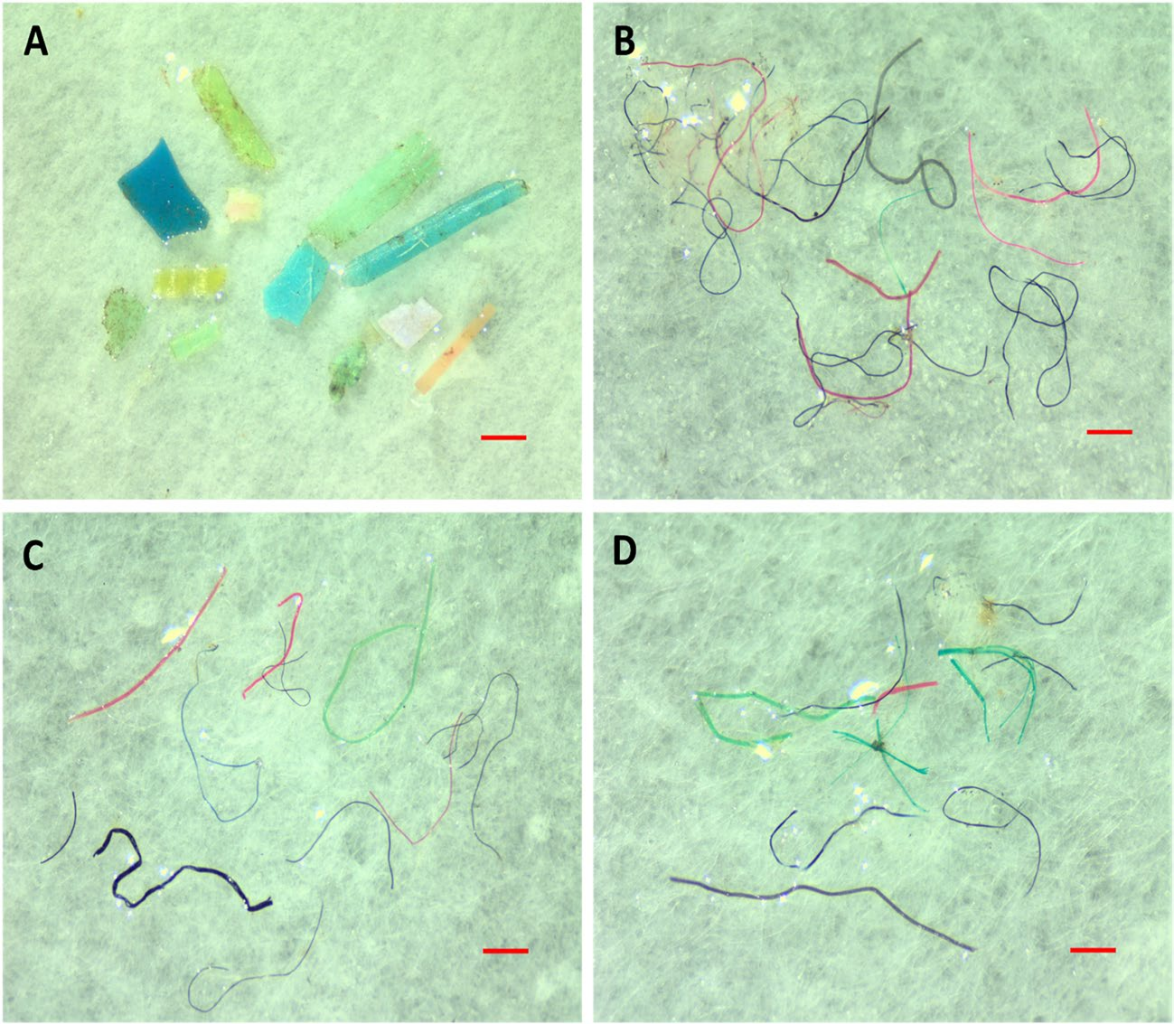
Photographs showing different shapes of microplastics obtained from sediment (**A** and **B**), *Chironomus* sp. (**C**), and chironomid tubes (**D**). (**A**) fragments and (**B**–**D**) fibers. The scale bar = 250 µm

**Fig. 4:**
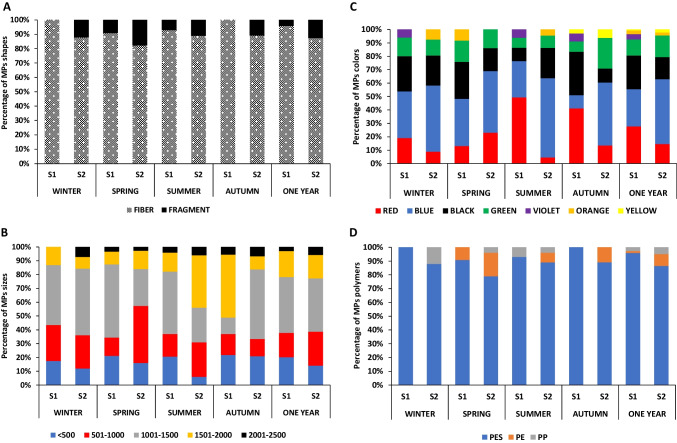
The percentage of the different microplastic shapes (**A**), lengths (**B**) with µm, colors(**C**), and chemical composition (**D**) collected from the sediment of the two wastewater sites

The MP shapes detected in the sediment of the two wastewater sites were fibers and fragments only (Fig. 3). Annually, fibers were the shape of the most prevalent particles observed in S1 and S2 accounting for 96% and 88%, respectively (Fig. 4A). Statistically, no significant differences in the abundance of fibers were recorded between **S**1 and **S**2 throughout the investigated year (*P* > 0.05). However, fragments were significantly higher in **S**2 than in **S**1 (*P* < 0.05).

Based on the length of sediment MP particles, MPs could be classified into five size classes: < 500, 501–1000, 1001–1500, 1501–2000, and 2001–2500 µm (Fig. 4B). The lengths of the fibers ranged from 684 to 2390 µm with an average of 1429 ± 495 µm and the fragments ranged from 157 to 1032 µm with an average of 554 ± 269 µm. Considering the width, the fibers ranged from 13 to 18 µm with an average of 14.6 ± 4 µm, and the fragments ranged from 64 to 632 µm with an average of 278 ± 181 µm. According to the MP size distribution during different seasons, the size in the range of 1001–1500 µm was the most dominant (39%), followed by 501–1000 µm (20%). According to the two wastewater sites, in S1, the most abundant lengths of MPs ranged from 1001–1500 µm, < 500 µm, and 1501–2000 µm (40%, 20%, and 18%, respectively); however, in S2, most MPs ranged from 1001–1500 µm, 501–1000 µm, and 1501–2000 µm (38%, 24%, and 17%, respectively) (Fig. 4B). Statistical analysis showed that the annual abundance of MP in the range of 1001–1500µm was significantly higher than that of the other MPs size classes (*P* < 0.05), and a significantly higher abundance of MPs size class < 500 µm was observed in **S**1 than in S2 (*P* < 0.05). However, MP size in the range of 501–1000 µm was significantly higher in **S**2 than in **S**1 (*P* < 0.05).

Figure 4C shows the distribution patterns of MP colors in the sediment samples. Fibers and fragments were introduced in a wide spectrum of colors, including red, blue, black, green, violet, orange, and yellow. Blue, red, black, and green colors observed in sediment samples accounted for 35%, 23%, 22%, and 13%, respectively, of the total MP particles. The distribution of MP colors displayed seasonal variation in the two sampling sites. Statistically, the red color proportion was significantly higher in **S**1 than in **S**2 (*P* < 0.05). However, the percentage of blue color was significantly higher in **S**2 than in **S**1 (*P* < 0.05).

**Fig. 5:**
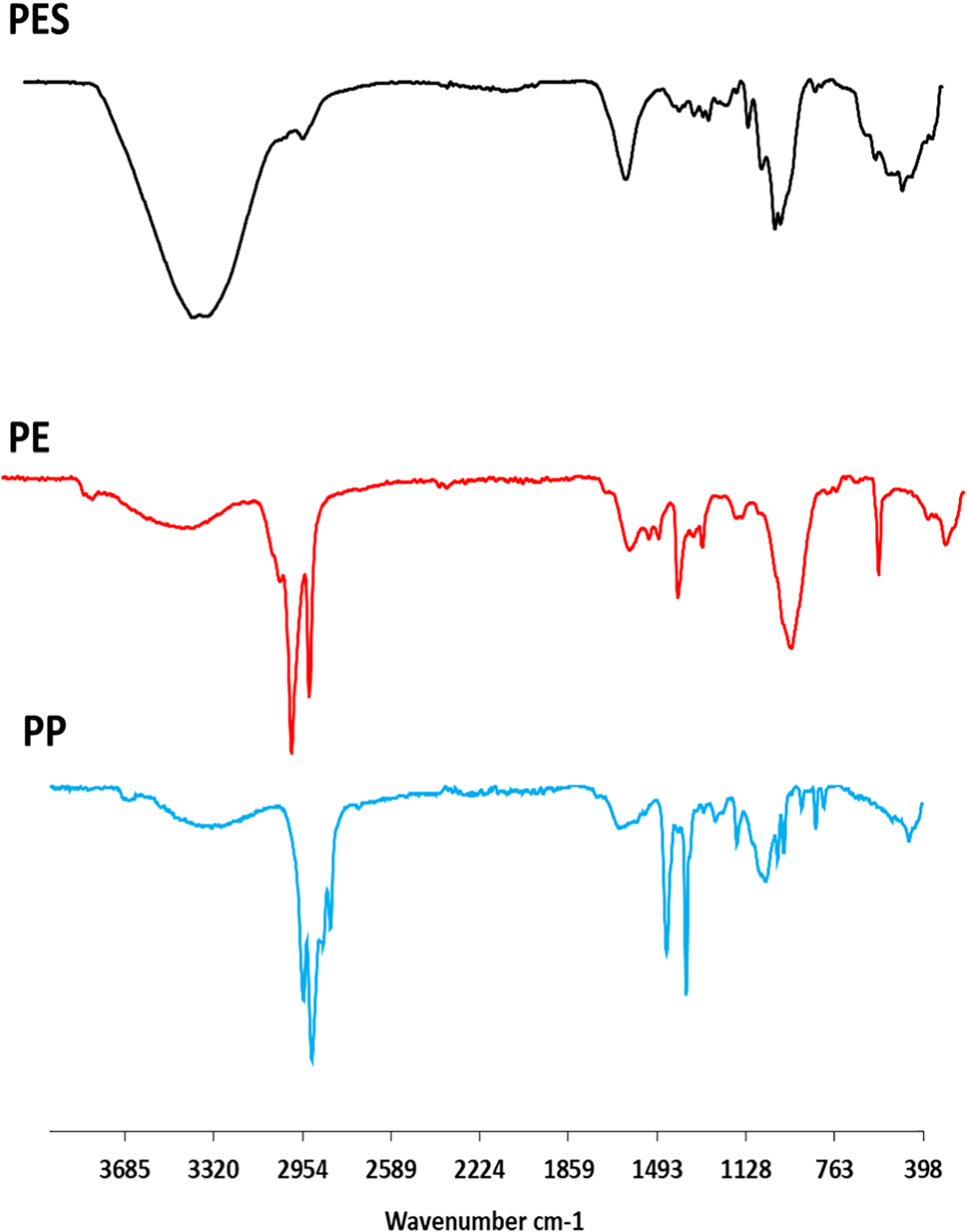
Micro-FTIR spectra of representative microplastic polymers extracted from sediment, *Chironomus* sp. larvae, and their tubes (PES, polyester; PE, polyethylene; and PP, polypropylene)

The MP particles collected from all sediment sampling sites in this study were analyzed by FTIR spectroscopy to identify common polymers. The following polymer types were identified in the sediment: polyester (PES), polyethylene (PE), and polypropylene (PP) (Fig. 5). Polymers of MPs in the winter, spring, summer, and autumn seasons were mostly PES (93%, 86%, 91%, and 94%, respectively), followed by PP in winter (7%), PE and PP in spring (14% and 2%, respectively), PP and PE in summer (5.5% and 3.5%, respectively), and PE in autumn (6%) (Fig. 4D). Significantly, a higher abundance of PES was found in sediment when compared with other polymers (*P* < 0.01). PP was significantly more abundant in winter and summer (*P* < 0.05), and PE was more abundant in spring (*P* < 0.05) than in the other seasons. Considering the two sites of wastewater, the most abundant polymers of MPs were PES (96% and 87%, respectively), followed by PP and PE in S1 (3% and 1%, respectively) and PE and PP in S2 (9% and 4%, respectively) (Fig. 4D). No significant differences in the abundance of PES were found among the two sites (*P* > 0.05) throughout the year. However, the abundance of PE and PP was more significant in S2 than in S1 (*P* < 0.05).

### MPs load indices

The PLI values of MP pollution during various seasons are displayed in Table 1. As can be observed in Table 1, calculated PLI values of the two wastewater sites over various seasons were intermediate, indicating a moderate pollution discharge level (hazard category II). The greatest and lowest PLI values were also recorded in the summer and spring, respectively. Additionally, in every season, PLI values were greater in S2 than in S1. Table 1 lists the H values for MP contamination at the two wastewater sites over various seasons. In Fig. 4D, the percentages of identified polymers used to calculate H are shown. Approximately equal percentages of each polymer were found in each of the four seasons. As a result, the obtained H values were nearly comparable between seasons. A medium danger to the environment is indicated by the H values of the polymers in various seasons, which fall under category III. This is due to the toxic properties of the polymers. In the two wastewater sites, the highest H of MPs was identified during the spring. Additionally, the MP RI index of sediment from the two locations indicated a modest degree of danger (degree II).

**Table 1:** Seasonal variations of microplastics impact indices in the sediments of the two wastewater sites

Seasons	Site 1 (S1)					Site 2 (S2)				
	$CFj$	$PLIj$	$H$	$Tj$	$RI$	$CFj$	$PLIj$	$H$	$Tj$	$RI$
Winter	193	14	403	1.16	223	212	15	400	1.05	223
Spring	141	12	463	1.8	259	174	13	519	1.7	290
Summer	215	15	420	1.04	223	263	16.5	449	0.96	251
Autumn	173	13	410	1.29	223	198	14	470.7	1.321	263
1 year	181	13.5 (medium)	416 (level III)	1.28	232 (level II)	212	15 (medium)	460 (level III)	1.21	257 (level II)

*CFj* contamination factor, *PLIj* pollution load index, *H* polymer risk assessment index, *Tj* toxicity coefficient of MPs, and *RI* potential ecological risk index

### Seasonal abundance and characterization of MPs in Chironomus sp. larvae and their tubes

#### Chironomus sp. larvae

**Fig. 6:**
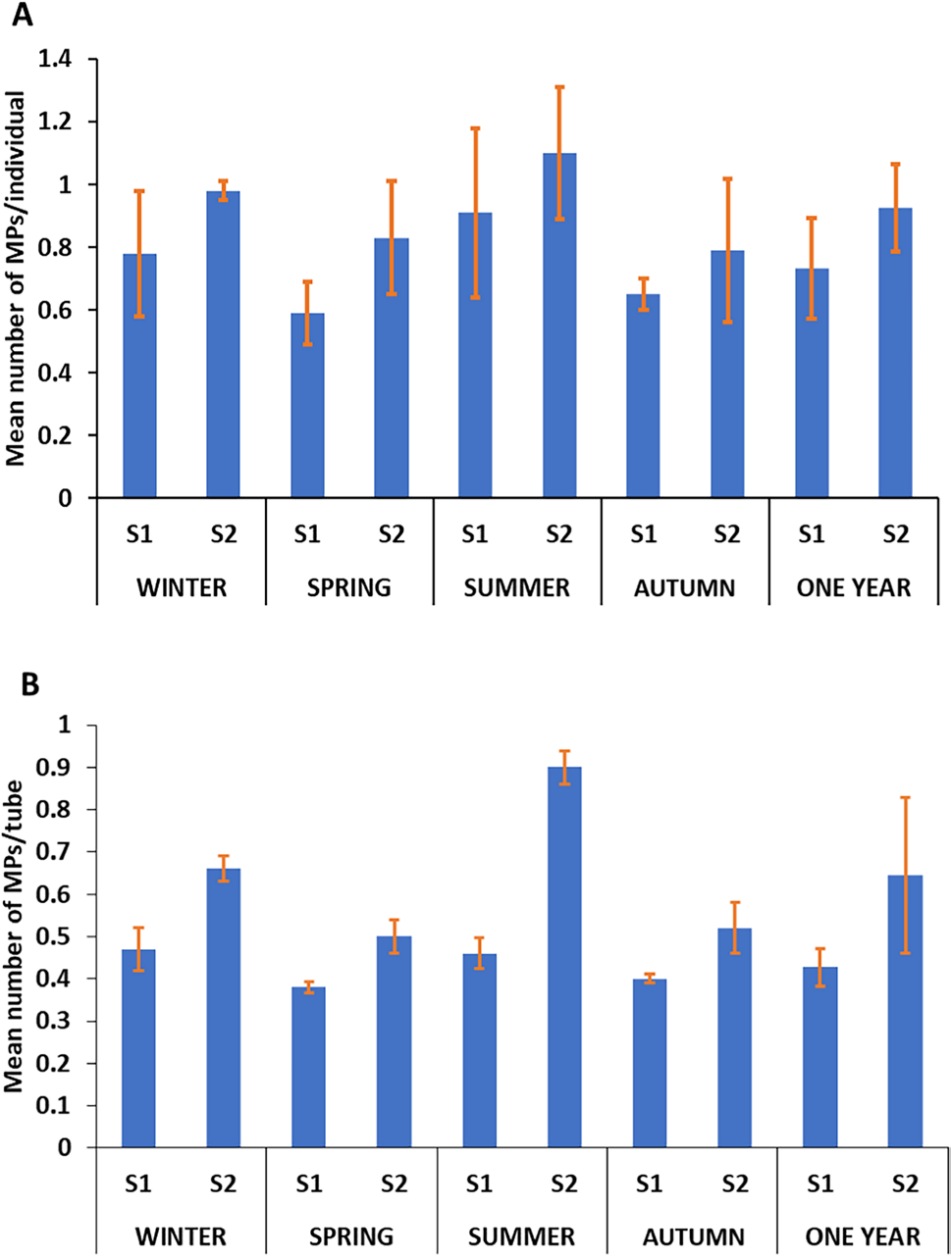
Mean seasonal abundance of MPs per individual of *Chironomus* sp. (**A**) and per chironomid tube (**B**) collected from the two sites of wastewater

**Fig. 7:**
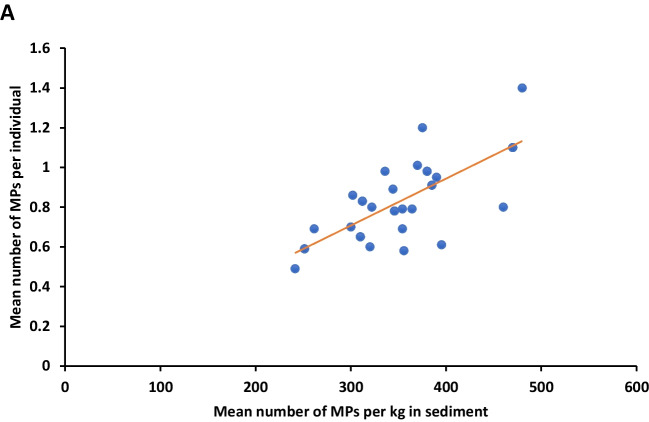
Relationship between the mean number of MPs per kg in sediment and per individual of *Chironomus* sp. larvae of the two wastewater sites

A total of 331 MP particles were extracted from 400 larvae across the two sites. The number of MP particles in S1 and S2 was 146 and 185, respectively. The maximum values of MPs per individual in S1 and S2 were observed in summer (0.91 ± 0.3 and 1.1 ± 0.3 particles/ind, respectively), followed by winter (0.78 ± 0.2 and 0.98 ± 0.028 particles/ind, respectively), but the minimum value of MPs in S1 was observed in spring (0.59 ± 0.1 particles/ind) and in autumn at S2 (0.79 ± 0.16 particles/ind) (Fig. 6A). Statistical analysis showed significant seasonal differences in MPs load per individual at both sites (*P* < 0.05). Moreover, the MP load per individual was significantly higher in S2 than in S1 in winter, spring, and autumn (*P* < 0.05). No significant difference was observed between the two sites in summer (*P* > 0.05). Regression analysis revealed a strong correlation between the number of MPs in the sediment and the number of MPs per individual larva in different seasons at both sites (*r* = 0.96, *P* < 0.05), as shown in Fig. 7. Higher significant seasonal differences in the size of *Chironomus* sp. larvae were found in S1 than in S2 (*P* < 0.05).

**Fig. 8:**
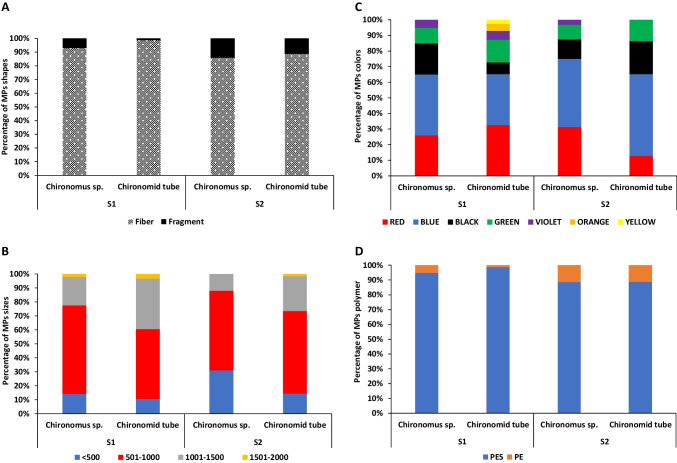
The percentage of the different microplastic shapes (**A**), lengths (**B**) with µm, colors(**C**), and chemical composition (**D**) collected from *Chironomus* sp. larvae and their tubes (chironomid tube) of the two wastewater sites

Most of the particles of MPs ingested by chironomid larvae were fibers and ranged from 86 to 93% of the total ingested MP particles in the two wastewater sites. The remaining proportions were fragments (Fig. 8A). The lengths of these fibers ranged from 522 to 1400 µm with an average of 840 ± 285 µm, while the fragments were between 86 and 94 µm with an average value of 90 ± 13 µm. Additionally, the MPs in the length range of 501–1000 µm accounted for the highest percentage (61%) (Fig. 8B). According to the MPs colors, blue was the most observed color (40.5%), followed by red (28%) and black (16%) (Fig. 8C). The dominant polymer in the larvae was polyester (89.5%) followed by polyethylene (10.5%) (Fig. 8D). The percentage of chironomid larvae contaminated with MPs was 95% and 100% in S1 and S2 of all samples collected during the study period, respectively.

### The relationship between MP size distribution across sediment and Chironomus sp. larvae

**Fig. 9:**
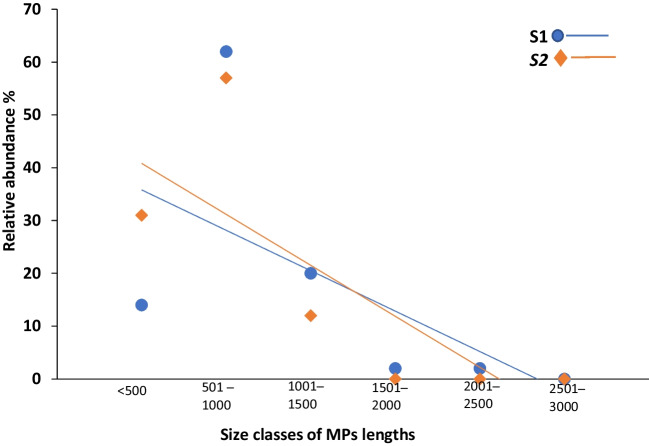
The relative abundance of MP size distribution across *Chironomus* sp. collected from the two wastewater sites

We performed a linear model analysis to examine the relationship between MP length distribution in sediment and that in chironomid larvae samples. Linear model analysis showed a linear relationship between MPs’ relative abundance and MP length class (Fig. 9). The length distribution of MPs detected in chironomid larvae demonstrated that MP concentrations decreased with increasing MP length. The percentage of MP < 1000 µm was 84% and 87% in the larvae obtained from S1 and S2, respectively. In addition to the evaluation of complete size distributions, we focused on the specific size class of 501–1000 µm, which may constitute a more favorable size for ingestion. As previously illustrated (Fig. 4B and Fig. 8B), the relative abundance of MPs particles sized 501–1000 µm was significantly higher in chironomid larvae than in their corresponding host sediments “S1 (*X*^2^ = 8.5, *P* < 0.05)” and “S2 (*X*^2^ = 4.7, *P* < 0.05)”.

### Effect of Chironomus sp. larval size on the abundance of MPs/individual

**Fig. 10:**
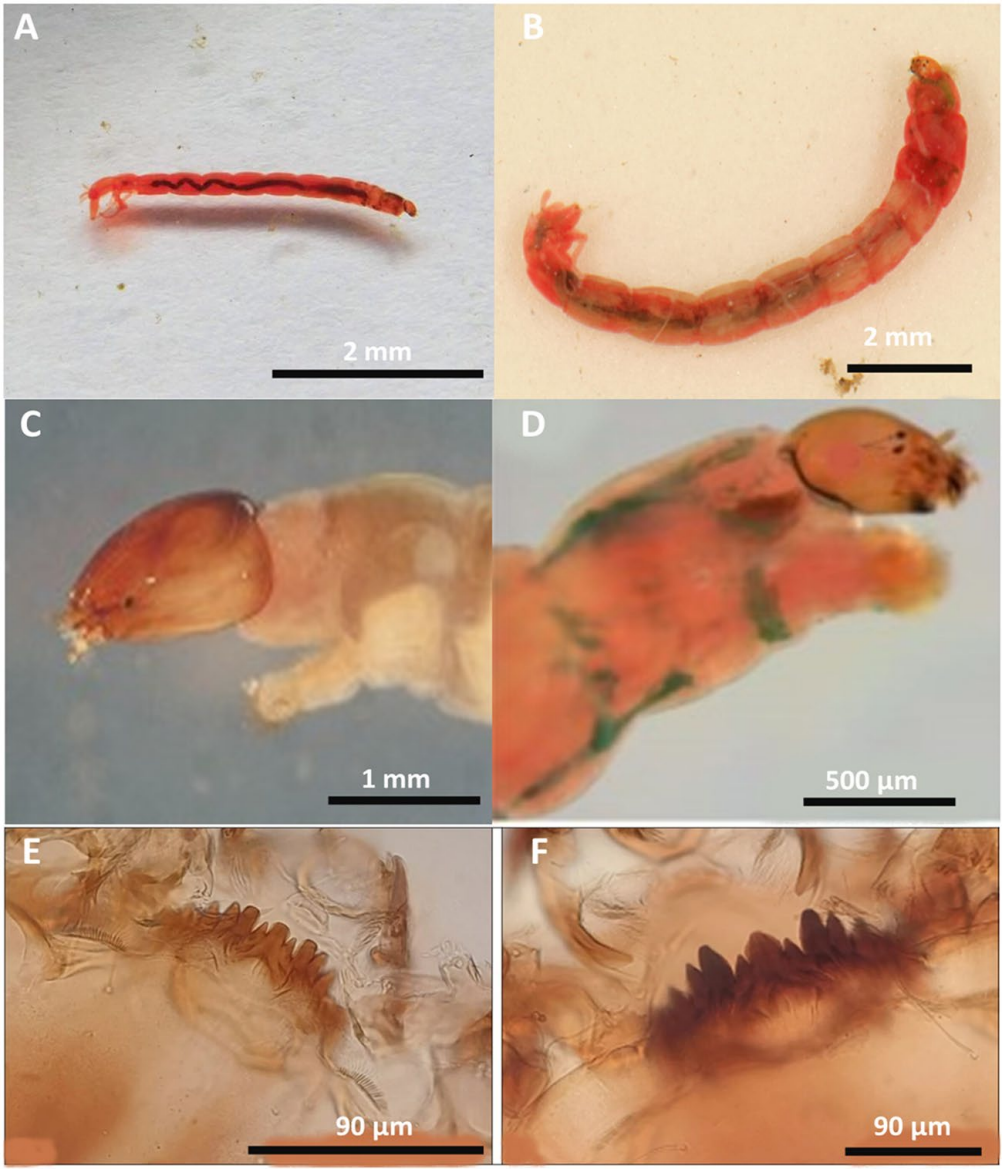
Whole body shape (**A**, **B**), head capsule (**C**, **D**), and mentum (**E**, **F**) of second (L2) and fourth (L4) instar larva of *Chironomus* sp., respectively

To evaluate how larval development affects MP uptake, a comparison between second (L2) and fourth (L4) instar larvae in terms of morphological characteristics was performed and is presented in Table 2. The data revealed that both body weight and length were larger in L4 than in L2 (Fig. 10). Moreover, L4 had a head capsule width of 0.55–0.74 mm with an average of 0.69 ± 0.15 mm and a corresponding mentum width of 0.158–0.176 mm with an average of 0.16 ± 0.01 mm, which was larger than those in L2. Statistically, the average body weight, body length, head capsule (width and length), and mentum width were significantly greater in L4 than in L2 (*P* < 0.05).

**Table 2:** Measurement of the weight (mg), body length (mm), head capsule width (mm), head capsule length (mm), and mentum width (mm) and characterization of MPs in the second (L2) and fourth (L4) instar larvae of *Chironomus* sp. (mean ± SD)

Stage	The mean weight of individual (mg)	Mean body length (mm)	Mean head capsule width (mm)	Mean head capsule length (mm)	Mean mentum width (mm)	Mean MPs particles/individual	The mean length of MPs (µm)
Second instar (L2)	4.8 ± 1.2	3.2 ± 0.5	0.21 ± 0.0.1	0.213 ± 0.022	0.059 ± 0.004	0.52 ± 0.11	Fibers 422 ± 84
Fourth instar (L4)	8.6 ± 2.4	14.8 ± 6.3	0.69 ± 0.15	0.37 ± 0.041	0.162 ± 0.013	0.91 ± 0.2	Fibers 937 ± 120
							Fragments 73 ± 20

Regarding MP load/larva, the number of MP particles/L4 was significantly higher (*P* < 0.05) than that in L2. Regression analysis revealed a significant positive effect of head width on MP count in *Chironomus* sp. (***r*** = 0.85, *P* < 0.05). Furthermore, fibers were the only type of MPs detected in L2 samples, ranging from 397 to 581 µm with an average length of 422 ± 84 µm. However, fibers and fragments accounted for 83% and 17%, respectively, in L4 samples. The fiber length ranged from 894 to 1132 µm with an average length of 937 ± 120 µm.

### Chironomid tubes

Figure 6B shows the seasonal abundance of MPs in chironomid tubes at S1 and S2. Chironomid tubes were observed during the investigated year in both wastewater sites. A total of 215 MP particles were extracted from 400 tubes across the two sites. The number of MP particles in S1 and S2 was 130 and 85, respectively. According to the number of MPs per chironomid tube, the highest values were observed in summer in both S1 and S2 (0.47 ± 0.05 and 0.9 ± 0.04 particles/tube, respectively). However, the lowest values were recorded in spring (0.38 ± 0.01 and 0.5 ± 0.04 particles/tube, respectively). It is important to note that the mean number of MPs per gram (w.w.) of the chironomid tube (particles/g) (data from the two sites) was 6.5 ± 2.3 particles/g. Significant seasonal differences in MP load per tube were observed in both S1 and S2 (*P* < 0.05). Moreover, the MP’s load was significantly higher in the S2 than that in the S1 in all seasons (*P* < 0.05). A strong correlation was recorded between the number of MPs in sediment and the average load of MPs in chironomid tubes at both sites (*r* = 0.87, *P* < 0.05).

Based on the type of MPs, fibers were the highest proportion and ranged from 79 to 100% of the total extracted MP particles, and the remaining proportion was fragments (Fig. 8A). The length of extracted fibers ranged from 603 to 1546 µm with an average of 962 ± 287 µm, while the fragments were between 187 and 1007 µm with an average of 597 ± 386 µm. The MPs in the size range of 501–1000 µm and 1001–1500 µm accounted for the highest proportion (54% and 30.5%, respectively) (Fig. 8B). According to the MP colors, blue was the most abundant (42%), followed by red (22.5%) and black (14.5%) (Fig. 8C). Polyester was the dominant polymer in chironomid tubes (93%), followed by polyethylene (7%) (Fig. 8D).

## Discussion

The main objective of this study is to highlight the seasonal abundance and characteristics variation of MPs in wastewater sediment of Sohag Governorate, Egypt that have not been reported yet. In addition, optimizing *Chironomus* sp. larvae and their protective structural buildings “chironomid tubes” as an effective indicator might reflect MP contamination. Considering the poorly managed WWTPs in developing countries (Mema 2010), they represent an important source of MP pollution in aquatic ecosystems (Chang 2015). WWTPs receive wastewater contaminated with MPs through domestic discharges including laundry wastewater and the uncontrolled discharge of industrial wastewater, in addition to landfill leachates (Wu et al. 2022). It is important to indicate that the study sites S1 and S2 are closely located to the main WWTPs in Sohag Governorate and continuously receive the discharged wastewater effluents. The results showed moderate seasonal abundance of MPs in both sites, while high values were recorded in summer (385.2 ± 38.2 and 470 ± 70 items/kg) in S1 and S2, respectively. Similar findings were recorded in the surface sediments of 28 stations in Sishili Bay, China (499.76 ± 370.07 items/kg d.w) (Zhang et al. 2019). However, the MP abundance was significantly higher in S2 than in S1 in all seasons of the year. This variation might be associated with the amount of sewage effluent discharged to the basins from the WWTPs. Among the dominant shapes of MPs observed in aquatic systems, fibers were the most dominant (Hajji et al. 2023). It has been reported that polyester-based cloths release over 1900 MP fibers in each single wash (Browne et al. 2011). This is consistent with our results revealing the high dominance of MP fibers in both WWTP sites accounting for 96% and 88% in S1 and S2, respectively. Considering most domestic wastewater of the majority of Sohag city citizens is discharged into WWTPs close to S1, while industrial wastewater mixed with domestic effluents is discharged into WWTPs near S2. That could explain the higher abundance of fibers in S1 than in S2. On the other hand, the strong relations between MP fragments and industrial activities (Jin et al. 2023) indicate why fragments were significantly higher in S2 than in S1. The sedimental MP fibers with lengths 1001–1500 µm are the most abundant in both sites (40 and 38%), in S1 and S2, respectively, concerning other length classes that agreed with the findings of Zhang et al. (2019). MP debris in the open environment is continuously subject to mechanical, chemical, and biological degradation (Andrady and Koongolla 2022) which could confirm the high percentage of small MP fibers detected in the current study. To obtain attractive plastic products corresponding with actual usage needs, plastic products are stained with different colors (Zhao et al. 2022). While environmental MPs appear in a wide variety of colors, blue-colored microplastics were the dominant color category (Athapaththu et al. 2020). In other findings, black, blue, and red are the most predominant colors (> 80%) (Montoto-Martinez et al. 2020). From this point, blue, red, black, and green MPs have 93% of the total MP particles collected from both sites. Notably, the consistency of MPs’ colors all over the world could reflect the popularity of plastic products and their globalization.

By FTIR, three polymers were identified: polyester (PES), polyethylene (PE), and polypropylene (PP) for the sedimental MPs. PES has a higher abundance in sediment when compared with other polymers in both WWTP sites. PES is one of the most important synthetic fibers that is widely used in a variety of other products including clothing and carpets, so it has the potential to release microplastics into the environment, especially during the manufacturing and cleaning process (Šaravanja et al. 2022). Therefore, domestic sewage derived from point sources plays an important role in MP pollution. While no significant differences in the abundance of PES in both sites, PE and PP were more significant in S2 than in S1. The wide uses of PE and PP in the industry (Li et al. 2021) confirm their higher abundance in S2. In a recent study, Ghani and coauthors found that MP fragments collected from the Red Sea belong to four plastic polymers, whereas PE and PP are the most common (Ghani et al. 2023). That could link the relative abundance of fragments with that of both PE and PP distributed in S2.

The risk assessment of MPs needs robust estimation of the characteristics, prevalence, distribution, and polymer types (Lindeque et al. 2020). Additionally, the wide variation in the abundance units of MPs led to the development of new standard parameters like pollution load index PLI, polymer risk index H, and potential ecological risk index RI. The pollution load index PLI is frequently used to explore the risk of MPs in sediment (Yin 2023). Our results revealed intermediate PLI values of the two wastewater sites over various seasons, while the greater values were recorded in the summer. Moreover, PLI values of S2 are greater than in S1 in correction with the higher abundance of MPs in this site. PLI indicated the variability of MP’s risk in different locations around the world which ranged from low (Neelavannan et al. 2022) to moderate risk (Kabir et al. 2022). In Egypt, PLI indicated moderate MPs pollution whether in the river Nile (Shabaka et al. 2022) or in the Red Sea (Ghani et al. 2023). Of note, the information submitted from abundance risk PLI is unreasonable without assessing the chemical composition of the collected MPs (Ding et al. 2022). Polymer risk assessment index H depends on the hazard score of each polymer, which greatly varies from one polymer to another (Lithner et al. 2011). Generally, the values of the H index refer to moderate risk (level III) of MPs distributed in both wastewater sites attributed to the high abundance of polyester of low hazard score (4). However, the relatively high value of the H index of S2 compared to S1 or in the spring of both sites corresponds to the increased percentage of polyethylene with 11 scores. However, the adverse effects of MPs on organisms and humans necessitate exploring their ability to internalize the organisms that inhabit such MPs-polluted environments.

Chironomids are widely distributed in wastewater sediments of Sohag Governorate (Khdre et al. 2023). Considering the high sensitivity and dominance of *Chironomus* larvae in polluted environments, they can be used to diagnose the ecological conditions variations in aquatic habitats. To such end, we examined the ability of *Chironomus* to reflect the environmental contamination with MPs at different seasons. Notably, our results showed that the mean number of MPs/individuals in chironomid larvae was significantly higher in **S**2 than in **S**1 taking a similar trend to sedimental MPs. Moreover, significant seasonal differences were observed in the MP loads within chironomid larvae at both sites. As a deposit feeder, there is a direct relationship between the accumulated MPs inside *Chironomus* larvae and those located in their environment (Nel et al. 2018; Khdre et al. 2023a). This relation was confirmed by regression analysis at different seasons of both sites. Since MPs accumulation has potential adverse effects on wildlife and humans (Xia et al. 2020), we observed growth inhibition of *Chironomus* larvae in S2 than in S1 proportionally with MPs abundance. A high abundance of MPs introduces more changes in substrate which in turn limits food uptake and therefore inhibits larval growth (Vos et al. 2002). In addition, MPs can fill the gut of *Chironomus* causing a negative energetic balance (Prata et al. 2023). However, malnutrition and sorbed contaminants of plastic additives are potential scenarios of impacts of MP accumulation (Narayanan 2023). As mentioned earlier for other freshwater invertebrates exposed to MPs, growth reduction was observed for *Gammarus pulex* (Redondo-Hasselerharm et al. 2018). To address whether MPs accumulated through development, MPs’ abundance inside two different larval instars was determined. The results revealed that the mean number of MPs/individual larvae in the fourth instar was significantly higher than that in the second instar. Also, MPs in the second instar larvae lacked fragments and had shorter fibers than those in the fourth instar larvae, which may be related to morphological restrictions in the size of the head capsule and functional mouthparts. These findings confirm the results of Redondo-Hasselerharm et al. (2018) who reported the preference in particle size and the quantity of ingested polystyrene particles changed throughout the development.

Shape, color, chemical composition, and other physical and chemical characteristics are primary factors that affect the hazards of MPs (Yin et al. 2023). Fibers were the main MPs type, accounting for 86–93% of the total MPs extracted in *Chironomus* sp. larvae, consistent with their higher abundance in the sediment of the two sampling sites. The regression analysis revealed that the relative abundance of MPs in *Chironomus* decreased as their size increased. Accordingly, the thinner width (13–18 µm) of fibers is a vital parameter stimulating their ingestion (Pirc et al. 2016). In this regard, previous studies reported the dominance of fibers in freshwater invertebrates (Naji et al. 2018; Akindele et al. 2019; Bertoli et al. 2022; Khdre et al. 2023b). On the other hand, it is necessary to pay attention to the detrimental impacts of MP colors on aquatic organisms (Chen et al. 2020). Considering the nonselective deposit-feeding of *Chironomus*, its larvae highly accumulated, blue-colored MPs (40.5%) compared to the other colors according to its abundance in the surrounding environment. It has been reported that daphnids are probably not able to distinguish algae from colored MPs (Chen et al. 2020). Nonetheless, blue-green algae are food resources for collector-gatherer groups (Parker et al. 2022), which may support high loads of blue color within the present larvae besides the dominance of blue MPs in their habitats. The length of fibers also affects their ingestion. *Chironomus* prefers low-length fibers (501–1000 µm) rather than the dominance fibers (1001–1500 µm) in the sediment. The limited dimension of the mouth apparatus and the difficulty in ingesting larger particles are the main factors that underline the small-sized microplastic selectivity of *Chironomus* (Prata et al. 2023). In addition, the ingestion of MPs through the alimentary canal is subject to variable conditions of physical and chemical digestion that lead to MPs-erosion and fragmentation (Sanchez-Hernandez 2021). The dominant polymer types accumulated in the *Chironomus* sp. larvae reflected the abundance of polymer types in the sediment (Munari et al. 2017). Therefore, *Chironomus* larvae accumulate polyester corresponding to its abundance in the host sediment. This suggests that *Chironomus* could be best employed as an MP qualitative bioindicator in freshwater. Considering the vital ecological role and important position in nutrient cycling that *Chironomus* has, any change in its distribution or physiological homeostasis will directly affect the higher organisms of the trophic web.

Some aquatic organisms create shelters to live inside using the available material in their environment. Since MPs are distributed in the surrounding environment, they could be used as building material (Nel and Froneman 2018). Polyvinyl chloride and polyester particles were incorporated into cases of the caddisfly (Ehlers et al. 2019). The accumulation of MPs in the tubes of *Chironomus* was not well highlighted. Herein, the seasonal variations of MP abundance in *Chironomus* tubes in both WWTP sites were detected. The results showed that MP abundance in C. tubes was significantly higher in **S**2 than in S1 and varied with the different seasons following the abundance pattern of MPs in sediment. Blue and red polyester fibers were the most abundant shape in the collected tubes, while a few percentages of the fragments were recorded following a similar pattern of sedimental MPs. Nonetheless, MP particles (54%) had a size range of 501–1000 µm, which were significantly higher than those observed in the sediment. This indicates the size preference of *Chironomus* larvae for constructing their tubes. Concerning the direct negative effects of MPs on organisms, MPs may lead to a reduction in shelter stability with an increasing number of MP particles (Ribeiro-Brasil et al. 2022). Hence, the shelter is easily transported far away by the water current, where MPs cause lightness compared with sand grains (Ehlers et al. 2020). Accordingly, *Chironomus*’ tubes could provide a picture round seasonal changes of MPs abundance located in their substrate as well as can be employed as a bioindicator for MPs distributed in freshwater.

## Limitations

The bioaccumulation of MPs and associated contaminants (such as heavy metals and persistent organic matter) within various higher trophic levels in various aquatic systems should be carried out to comprehend the implications and risks of MPs as well as the toxicity caused by the adsorption presence of these contaminants in freshwater biota.

## Conclusion

Even though MP contamination is a worldwide problem, research on the spatiotemporal distribution of freshwater MPs is still in its infancy. This is the first study to consider both human activities and seasonality in connection to internal MP contents in two wastewater sites in Egypt’s Sohag Governorate. Our findings support a prior study that revealed that *Chironomus* sp. might be beneficial as a freshwater MP bioindicator. Furthermore, the current study is the first to document the use of chironomid tubes as indicators for freshwater MPs. It can serve as a warning sign for MP buildup in all compartments of the aquatic environment. Furthermore, our findings were intended to provide information regarding the seasonal variation in MPs in wastewater, hence increasing the understanding of the seasonal effect on MPs pollution and risk levels in freshwater ecosystems. Consequently, our findings will help policymakers and the government, in partnership with international organizations, implement suitable management strategies to minimize the waste of plastic. Our results found that blue polyester fibers are much more prevalent than other polymers, colors, and shapes of MPs, and S2 was more highly contaminated with MPs than S1 during the four seasons of the year. Additionally, the abundance of MPs/individual was higher significantly in the fourth instar larvae (*P* < 0.05) than in the second instar. Further studies on the applicability of chironomid tubes as MP bioindicators in various freshwater environments throughout the world should be taken.

## Data Availability

All data analyzed in this manuscript is included in the published article.
